# The open gym—an active (lunch)time offering at all-day schools

**DOI:** 10.3389/fspor.2025.1543771

**Published:** 2025-03-25

**Authors:** Ilaria Ferrari, Patricia Schuler, Johanna Kress, Kathrin Bretz, Lukas Niederberger

**Affiliations:** ^1^ Research Group Exercise and Sport, Zurich University of Teacher Education, Zurich, Switzerland; ^2^ Centre for Teaching Professions and Continuing Professional Development, Zurich University of Teacher Education, Zurich, Switzerland; ^3^ Sports Office City of Zurich, Competence Center Physical Education, Zurich, Switzerland

**Keywords:** all-day schools, physical activities, health promotion, open gym, extended education, quality extracurricular programme

## Abstract

**Introduction:**

The implementation of “all-day schools” (schools with extended educational programmes) provides pupils with the opportunity to engage in a multitude of different activities and learning contexts throughout the day, in addition to their core, compulsory curriculum. These activities may include a diverse range of sports-oriented activities at regular intervals throughout the school day and are accessible to all pupils, irrespective of gender and socio-cultural background. In the context of the project, “Sport in the School Environment” various extended physical activity programmes were implemented and evaluated in 14 primary all-day schools in Zurich (Switzerland) between 2019 and 2021. This article focuses on the extended pedagogical physical activity of the “open gym,” a free physical activity programme during the lunch break in all-day schools and examines how the open gym is utilised by different groups of pupils in relation to gender and socio-cultural background.

**Methods:**

Data from 401 s-grade pupils were collected using a standardised questionnaire. The participation of pupils in the different programmes was analysed using descriptive statistics, and the relation between the programmes and the socio-cultural background of the pupils was determined by a chi-square test for nonparametric data.

**Results:**

Overall, 30%–40% of the children participated in different extended educational programmes, with boys participating more frequently than girls. The various activities however, also engaged girls and increased their participation. The initial findings indicate that the pupils made active use of the open gym, with a higher frequency of attendance among boys compared to girls.

**Discussion:**

The results indicate that the open gym is a significantly utilised programme (37% of children participated) and is frequently accessed especially by boys from a variety of different socio-cultural backgrounds.

## 1 Introduction

In recent years, all-day schools have been introduced in German-speaking Switzerland. In the city of Zürich, these are schools which offer an extended educational programme before the beginning of the official school day in the morning and after the end of the school day in the afternoon, including leisure time during lunchtime and in the afternoon break. The expansion of schools' extended educational programmes increases the length of time pupils and young people spend at school. The daily life of children and young people in urban areas in German-speaking Switzerland is increasingly characterised by (all-day) school structures, which reduces the timeframe in which children and adolescents can organise their time freely (and outside of school) (1). The increasingly institutionalised daily life of children and young people, which has also been criticised for the scholarisation of leisure time (2), is accompanied up by an expansion of the state's mandate to provide care and education (3) and an increased responsibility on the part of the school to foster physicality and physical activity in children and young people as they grow up (4, 5).

According to Kemmis (6) education plays a critical role in shaping individuals and the formation of the cultural, material, and social conditions of our collective life (6). All-day schools, therefore, have the responsibility for and the potential to develop and implement adequate and varied physical activities for children and young people (7, 8) in order to improve the quality of school life. This is an key concern, as the daily lives of many students are characterised by a lack of physical activity (9, 10). The all-day school programme offers an opportunity to stimulate interest in and enjoyment of physical activity early in life through individualised programmes, and to engage children who may not have access to an active lifestyle at home (11). Physical education classes do not provide enough opportunities for children to meet the guidelines on the recommended amount of moderate to vigorous physical activity, therefore other forms of physical activity, such as extended educational sports programmes in schools should be implemented (12). Schools are important settings for promoting physical activity among young people (13). In practice, extended educational sports programmes may include structured sports programmes after lessons, supervised physical activities at lunchtime, and free physical activities during breaks as well as before and after lessons (4). Extended educational sports programmes are situated at the interface between compulsory physical education and training at a club, between formal, non-formal, and informal learning opportunities, and between leisure and education (3, 8, 12, 14). In contrast to physical education, which is compulsory and follows a prescribed curriculum, extracurricular programmes are typically locally-designed and participation is voluntary. These programmes also differ from sports clubs and interscholastic programmes in that, for example, they are not selective in terms of participants, do not focus on a single competitive sport, and are primarily designed to provide ongoing childcare and supervision rather than to teach a specific skill or performance (12). The provision of an infrastructure offering experiences and opportunities for physical activities introduces pupils to new types of physical activity, thereby facilitating a movement-oriented rhythm within the school day (4). In Switzerland, compulsory education extends over a period of eleven years. Children typically begin preschool at age four, which forms part of the mandatory primary education system. After two years of preschool, children enter primary school in the first class, for a duration of six years. Afterwards, pupils progress to secondary education, enrolling in either a grammar school or a general secondary school. The secondary education phase lasts for an additional three years. Compulsory schooling concludes at the end of grade 9. The primary education curriculum is divided into two cycles: cycle 1 (preschool to second grade) and cycle 2 (third to sixth grade) (15).

The project “Sport in the School Environment” [German: Sport im Lebensraum Schule], funded by the Federal Office of Sport (BASPO, Bundesamt für Sport) aims at developing, implementing, and evaluating extended physical educational opportunities at 14 public primary schools in the city of Zurich. The schools are located in different and diverse neighbourhoods. Extended physical educational activities were implemented before and after the school day in the academic years 2019/20 and 2020/21. These activities were provided free of charge and were accessible to all students of the school. The physical activities were trialled by the pupils for a period of two years with the objective of increasing physical activity as a meaningful leisure activity within the school environment, in accordance with the principles of holistic and sustainable health promotion (10, 16).

An opportunity model can provide a theoretical basis for the evaluation of extended educational physical activities (17, 18). Who is using the provided opportunities and to what extent? In the context of extended educational activities, the fundamental dimensions of school quality must be complemented and refined with the incorporation of specific standards and objectives that are anchored in the principles of social pedagogy or youth work (19). The effectiveness of the programme depends not only on the quality of the programme itself but, due to its non-mandatory character, on its use by the pupils. Traditionally, the component of use also includes the duration, intensity, and regularity of participation. Therefore, students' participation profiles play an important role in defining the quality of the non-formal learning activity (20, 21).

A comprehensive School Physical Activity Programme, comprising four extended educational programmes, was proposed to the participating schools: (1) Free sports courses (year-long in duration) in the late afternoon at the conclusion of the formal school day (from 3:30 to 4:30 p.m.); (2) the equipment box for movement during recess; (3) three different mobile facilities; and (4) the open gym during lunchtime.

The sports courses comprise structured activities, such as soccer or dancing, as well as multi-sport courses that require parents to register their children for and which are available to pupils free of charge throughout the school year. The year-long course takes place once a week.

The equipment box contains varied pieces of sports equipment, such as balls, ropes or skateboards which pupils can use during their designated recess periods for recreational activities. The box is typically situated in the school playground.

All schools participating in the project had the opportunity to request the provision of mobile facilities, which were installed in the school playground (22), typically for a period of four to eight weeks. Three facilities were offered:

• The “skatepark” comprised modular park elements for cycling and roller sports, which could be used with a variety of cycling and roller vehicles (e.g., BMX bikes, kickboards, skateboards, inline skates, etc.).
• The “street soccer” playground was a perimeter field equipped with weighted plates and a ground anchorage, including a net catcher. This facility allowed for the playing of soccer and floorball.
• The “pump track” was a wave-through track constructed from prefabricated elements for cycling and roller sports. The track allowed for the use of a variety of equipment including mountain bikes, BMX bikes, kickboards, skateboards, and inline skates.

The open gym represented a low-threshold opportunity for physical activity in the context of extended education which pupils could utilise spontaneously and typically without prior registration, during their lunch break. The open gym was organised differently depending on the needs of the individual schools and the skills and qualifications of the staff. At some schools, caregivers supervised the open gym during lunchtime and provided activities for games or movement parcours. Furthermore, the open gym could be divided into discrete sections, with activities such as floorball or football taking place concurrently with free play. To ensure the safety and supervision of the open gym, the Sports Department of the City of Zurich provided further education for the caregivers, with a particular focus on safety and equipment handling. In a smaller number of schools, the open gym was supervised by qualified coaches, including licensed physical education teachers with either a bachelor's or master's degree in physical education, or club trainers with extensive experience and a trainer's licence.

The project aim of the open gym was to offer voluntary and free physical activities for pupils that established the rhythm of their weekly and daily routine with an alternation between phases of movement and rest, physical activity, and concentrated learning, in which the pupils were allowed and encouraged to take the initiative and could cultivate peer relationships (23). The open gym was characterised as a non-formal and informal learning opportunity in an extended educational framework at all-day schools, offering space and opportunities for interdisciplinary and subject-specific learning and thus meeting the quality criteria for the successful implementation of opportunities to explore autonomy, support, participation, recognition and everyday life orientation across the school day (24–26).

This article addresses two central questions concerning the use of extended educational sport-oriented opportunities, particularly in relation to the open gym:

1. How do pupils use the open gym programme in comparison to the other extended educational physical activities?
2. How is the open gym used by various groups of pupils in all-day schools as related to gender and socio-cultural background?

## 2 Materials and methods

The Physical Activity Programme, comprising four extended educational offers was implemented in the academic years 2019/20 and 2020/21. The COVID-19 pandemic did not severely impact the study's implementation. While Swiss schools remained closed for eight weeks in spring 2020 (March 16–June 8), and physical activities were initially restricted to small outdoor groups, the survey was postponed until immediately prior to the summer holidays. From the 2020/21 school year onwards, all physical activities, including the open gym, resumed with regularity, albeit with precautions such as mask-wearing and the exclusion of contact activities. All second-grade primary school pupils of the participating schools were surveyed in spring summer 2021 about their use of the extended educational offers implemented at the schools by means of a standardised paper questionnaire (27). The study was conducted in accordance with the Declaration of Helsinki. The Zurich City School Office and the school principal provided their consent for the survey to proceed. The questionnaire was completed in a classroom setting, and parental consent was not required. Pupils' participation in the survey was entirely voluntary.

The standardised questionnaire included questions on participation in the open gym, equipment box, mobile facilities and participation in the sport courses. Additionally, based on the findings of Quellenberg (28), we enquired about the pupils’ utilisation of the aforementioned facilities and additionally about their personal background. Furthermore, we included questions about the language(s) spoken at home, as documented by Ferrari et al. (22).

In response to the question, “Which extended educational physical activities do you attend each year?” the respondents could indicate the chosen course or “no courses.” In response to the question, “What else do you attend?” they could select the following options: “structured sports classes,” “pop-ups,” the “equipment box,” and “open gym.” The pupils' age, gender, and the language(s) spoken at home were asked. The language(s) spoken at home is considered as a reliable indicator of pupils' socio-cultural background (29). Pupils who do not Swiss German at home indicates some degree of a migratory background. However, it should be noted that other factors, such as family structure, milieu, socioeconomic status, and type of linguistic socialisation, also have an impact on the everyday framework for the lifeworld of pupils with a migration background (29). In the questionnaire, options such as “Swiss German/High German/French/English/Italian/Others” were available. For data analysis, the answer “Others” was grouped with “French,” “Italian,” “High German,” and “English” to “Other languages”. Although there are four official languages in Switzerland [(Swiss)German, French, Italian and Romansh], only the distinction between Swiss German and other languages was made, given that the local spoken dialect in Zurich, where the data collection took place, is Swiss German (30). The decision to divide the participants into two language groups was taken in order to minimise differentiation and for reasons of research feasibility. The socio-cultural background of the children was linked to the variables “Swiss German language” and “Other languages”, based on the explanations given about the specific regional language spoken in the study region and by considering language-specific curricula that exist in Switzerland, which influence the school setting. This dichotomy indicates whether the children's family is deeply rooted in the regional culture, or whether the family speaks another language and thus is likely to have migrated from overseas or from another language region of Switzerland and therefore provides a different linguistic and cultural context. In addition, this differentiation is based on studies that have demonstrated a strong correlation between language region, and physical activity and motor skills in young children. Regional influences were identified across countries and within countries among Swiss children (31, 32). In addition, Lamprecht and colleagues (33) found that children's physical activity is influenced by their migration status. The participation in sport in different settings of girls and boys and of national and international origin differs. Specifically, the total time spent doing physical activity time by children of international origin is more closely linked to the school setting than the one of national-origin children, who show a higher proportion of total physical activity time in sports outside of school (33).

### 2.1 Sample

The survey was conducted with a total of 401 s-grade pupils from 14 schools. In total 226 boys and 175 girls with an average age of 8.1 (±0.42) years (Table 1) completed the questionnaire. The language spoken at home by 221 (55.1%) pupils was Swiss German and 215 (53.4%) pupils spoke other languages. Approximately half of the boys spoke Swiss German at home; the remaining pupils spoke other languages; approximately two-thirds of the girls spoke Swiss German at home and approximately half spoke other languages (multiple responses possible; see Table 1). It should be noted that the pupils were able to give several answers to the language question. In total, 73 pupils (40 boys and 33 girls) stated that they spoke both Swiss German and another language at home.

**Table 1:** Description of the sample of second-grade pupils (*n* = 401).

		Age	Language Swiss German	Other languages
2nd grade	*n* (%)	M ± SD	*n* (%)	*n* (%)
Total	401 (100)	8.1 ± 0.4	221 (55.1)	214 (53.4)
Boys	226 (56.4)	8.1 ± 0.5	113 (50.0)	129 (57.0)
Girls	175 (43.6)	8.1 ± 0.4	108 (61.7)	85 (48.6)

### 2.2 Data analysis

The statistical analysis of the questionnaires was carried out with SPSS Statistics 28 software for Windows (IBM Corporation, Armonk, USA).

Descriptive statistics were employed to illustrate the participation of pupils in the various programmes, including open gym, sports courses, mobile facilities, and equipment boxes. To investigate how many pupils participated in only one or several offers, we created a new variable. The data pertaining to the mobile facilities and the equipment box were aggregated in a new variable, designated “unguided/free offers.”

For further statistical analysis, we focused on the open gym and the sports courses, as these were the most frequently utilised programmes. Differences between the various programmes and the spoken language were determined by the chi-square test for nonparametric tests. The significance level was set at *p* ≤ .05. As we had a degree of freedom of 1 (df = 1), we applied the Yates correction for the chi-square test. To measure the strength of the association between two variables the effect size Cramér's V was employed. The values of Cramér's V indicate a small effect within the range of .07–.21, a value of a medium effect is within the range of .21–.35, and a value larger than.35 indicates a large effect (34).

## 3 Results

In relation to the total sample (*n* = 401), 149 (37.2%) pupils of the different schools used the open gym, 169 (42.1%) of the pupils participated in the sports courses, and 126 (31.4%) used the equipment box during recess. The mobile facilities were not available at all schools, and they were used by 91 (26.8% rel. on a total of 340) of the 340 pupils who had access to use them. A total of 84 pupils (20.9%) did not utilise any programmes (Table 2).

**Table 2:** Use of the physical activity program with four extended educational offers by the second-grade pupils (*n* = 401, or *n* = 340 for the mobile facilities).

	Open gym	Year-long courses	Mobile facilities (*n* = 340)	Equipment box	No offers
	*n* (%)	*n* (%)	*n* (% rel.)	*n* (%)	*n* (%)
Total (402)	149 (37.2)	169 (42.1)	91 (26.8)	126 (31.4)	84 (20.9)
Male (226)	100 (44.2)	114 (50.4)	58 (17.1)	69 (30.5)	35 (15.5)
Female (175)	49 (28.0)	55 (31.4)	31 (9.1)	57 (32.6)	49 (28.0)

As seen in Table 2, all physical activities were generally used more by boys than girls, even though the equipment box was used almost equally by boys and girls. In relation to the total number of boys (*n* = 226), 50.4% (*n* = 114) participated in the sports courses, 44.2% (*n* = 100) used the open gym, 30.5% (*n* = 69) used the equipment box, and 26.8% (*n* = 91) used the mobile facilities. With regard to the female cohort (*n* = 175), 31.4% (*n* = 55) participated in the sports courses, 28% (*n* = 49) used the open gym, 32.6% (*n* = 57) used the equipment box, and 9.1% (*n* = 31) used the mobile facilities (Table 2).

Approximately one-fifth of pupils did not use or participate in any of the offers (20.9%; Table 2). Furthermore, the results in Table 3 show that 14.4% of the pupils attended unstructured/free activities which represents the combination of mobile facilities and the equipment box. These activities were supervised but not structured and not directly assisted by caregivers or teachers. The pupils were provided with the requisite equipment and infrastructure to facilitate movement. 11.7% of the pupils used the open gym and 8.9% of the pupils attended the year-long courses. 10% of pupils accessed all three main programmes in different combinations.

**Table 3:** Number of pupils attending the various offered physical activities.

	Number	% of pupils relative to 360
Only unguided/free offers	52	14.4
Only open gym	42	11.7
Only sports courses	32	8.9
Unguided/free offers & open gym	36	10.0
Unguided/free offers & sports courses	43	11.9
Open gym & sports courses	35	9.7
Unguided/free offers & open gym & sports courses	36	10.0
No offers	84	23.3

The percentages are based on a total of 360 pupils, as some data could not be included in this Table due to missing data of pupils, who did not answer this question.

Table 4 shows language as a socio-cultural variable regarding the use of the of the open gym and sports course programmes. To analyse relationships between language and the programmes, a chi-square test (*χ*^2^) was performed between languages other than Swiss German and participation in the open gym, as well as between other languages and participation in sport courses. In the analysis, no expected cell frequencies were less than 5.

**Table 4:** Descriptive statistics of spoken language and participation in the open gym or the year- long sport courses.

	Open gym		Year-long courses	
	Swiss German	Other languages	Swiss German	Other languages
	*n*	*n*	*n*	*n*
Total	74	97	138	113
Boys	45	71	65	56
Girls	29	26	73	57

Children could indicate more than one language spoken at home, for this reason, some children may appear in both categories.

The analysis of languages other than Swiss German spoken at home by the pupils showed that speaking other languages was significantly associated with increased participation in both the open gym [*χ*^2^(1, *n* = 373) = 14.470 *p* < .001, Cramér's V = 0.202] and in the sports courses [*χ*^2^(1, *n* = 397) = 4.568 *p* = .033, Cramér's V = 0.112]. Similar correlations were found only for boys when the analysis was repeated separately for boys and girls. In the separate analysis for the girls, no significant correlations were found. For boys, the relationship between other languages and participation in the open gym represented a medium association with Cramér's V = 0.281, *χ*^2^(1, *n* = 209) = 15. 377 *p* < .001, whereas for the sport courses and other languages, the association was small, Cramér's V = 0.148, *χ*^2^(1, *n* = 225) = 4.324, *p* = .038. Closer analysis of the open gym demonstrated that boys and girls who spoke Swiss German at home and girls who spoke languages other than Swiss German predominantly did not participate in the open gym (girls and boys who speak Swiss German represented 15.5% of all pupils and girls speaking other languages 13.5%). On the other hand, more than half of the boys who spoke other languages than Swiss German attended the open gym (17.7% of all pupils).

The same analytical methodology as that employed in Table 5 was applied to the Swiss German language and other languages. A statistically significant correlation was found between the Swiss German language and participation in sports courses *χ*^2^(1, *n* = 399) = 4.243 *p* = .039. The data indicated that pupils who spoke Swiss German were less likely to participate in the sports courses. However, the strength of the association between these variables was relatively weak (Cramér's V = 0.108). The mentioned significant relationship was present only for boys [*χ*^2^(1, *n* = 226) = 5.115 *p* = .024, Cramér's V = 0.159] when boys and girls were considered separately. No significant correlation was identified between the Swiss German language and participation in the open gym.

**Table 5:** Relationships between the spoken language (other languages than Swiss German) and participation in the open gym or the year-long sports courses.

			Other languages		*χ* ^2^	Cramér's V *φ*c
			yes	no		
			*n* (%)	*n* (%)		
Total	Open gym	yes	97 (26)	51 (14)	14.470**	0.202
		no	101 (27)	124 (33)		
	Year-long courses	yes	101 (25)	66 (17)	4.568*	0.112
		no	113 (29)	117 (30)		
Boys	Open gym	yes	71 (34)	29 (14)	15.377**	0.281
		no	47 (23)	62 (30)		
	Year-long courses	yes	73 (32)	40 (18)	4.324*	0.148
		no	56 (25)	56 (25)		
Girls	Open gym	yes	26 (16)	22 (13)	0.513	0.069
		no	54 (33)	62 (30)		
	Year-long courses	yes	28 (16)	26 (15)	0.072	0.033
		no	57 (33)	61 (36)		

* = *p* < .05.

** = *p* < .001.

## 4 Discussion

The expansion of non-formal learning opportunities through extended educational programmes represents a central feature of all-day schools. Various forms of physical activities in all-day schools are available before, after, and between regular lessons and differ in terms of content and subject matter as well as the degree of self-determination granted to the pupils. Certain physical activities resemble regular sports lessons because they are organised similarly in terms of time frame and content. Other programmes are less formalised and can be used by the pupils without any registration requirements. Such programmes are supervised by a teacher or a trainer but are significantly less controlled and regulated. One of these less regulated and formalised programmes is the open gym during lunchtime. Although teachers or trainers are present in the gym, they do not teach any prescribed physical education content. The pupils are allowed to determine both what and with whom they wish to play. This programme differs significantly from physical education and special extended educational programmes and forms an important building block for the quality of leisure activities at school (35, 36). On average, Swiss children and adolescents spend 61% of their day engaged in sedentary behaviour (37). The results show that participation in the programmes has the potential to improve the amount of time children are physically active for. Pupils determine for themselves the extent to which they wish to participate and engage actively. It is possible to observe from the sidelines, to participate or to take the initiative, suggesting and initiating a game. In this way, the pupils receive opportunities for involvement, participation, initiative, and self-determined interaction with other pupils. This, of course, also involves negotiating processes and repeated discussion, coming to a consensus on the different aspects of the activity, and defining common rules within the gym hall context. The active use of the open gym indicates however, that this format is appealing to pupils.

The Physical Activity Program presented here was used by boys more often than by girls. Speaking languages other than Swiss German at home was significantly related to increased participation both in sports courses and in the use of the open gym. Pupils who did not speak Swiss German used the sports offers at school much more intensively than pupils who did speak Swiss German. Since language is correlated with migration background, this could give an indication of a migration background of the pupils. Finding that pupils with a migration background are more likely to use the Physical Activity Program and especially the open gym is a remarkable result of this study, especially when considering that pupils with a migration background are more likely to be physically inactive (33, 38). In addition, language can be a barrier for children, as children who do not speak Swiss German may have fewer opportunities to connect with others in sports clubs or during leisure activities. Therefore, school and extracurricular programmes could act as an effective mechanism to connect pupils of this demographic with increased physical activity. Children who speak Swiss German at home attend the school activities in leisure time less, but are more actively involved in sports clubs. According to (39) and (40), participation in club sports between children and adolescents with and without a migration background depends on parental education and employment status. Families with low incomes tend to be less active during their leisure time (41). Socioeconomic differences were particularly evident in club sports compared to outdoor physical activity. Adolescents from lower social classes have, in some cases, less access to structured exercise and sports programmes but spend more time engaging in active play (42–44).

Our analysis of The Physical Activity Program demonstrates that it reaches and encourages pupils with a migration background to take part in sports and physical activities, as they are available in a low-threshold context, represent an attractive complement to the classroom lessons, and provide space for initiative and self-determination (21). This leads to a holistic promotion of physical activity and health, which is of great importance for the pupils' further development (10). Neuber and Züchner (45) reported similar patterns of use and interpreted them as a compensatory effect of all-day schools for fostering physical activity (46). The influencing factors on physical activity outcomes are known to be many and diverse, but in early childhood, the family context plays a key role (47). Depending on the socio-cultural background and parental decision-making, pupils receive more or fewer opportunities for sports-related experiences to shape their sporting activity (39). Furthermore, one of the advantages of implementing the Physical Activity Program in all-day schools, is that all pupils can be reached, regardless of their socio-cultural background, which in turn fosters social interaction between all demographic groups in the school.

### 4.1 Limitations

The multi-method data collection and analyses presented in this article provide a preliminary insight into the use of the open gym. Further studies with a longitudinal perspective must be conducted to accurately identify linking mechanisms between curricular and extended education. Furthermore, the influence on and development of physical health and social skills should be measured with a longitudinal quasi-experimental assessment of objectively measured physical activity.

As discussed in the Methods section, due to the COVID-19 pandemic, Swiss schools were closed for eight weeks in the spring of 2020 (from March 16 to June 8), after which all physical activity was conducted in small groups and outdoors until the summer holiday of 2020. The survey was however, postponed until shortly before the summer holidays to allow for a continuous 5-week period in which the extended activities, e.g., the open gym were operable. Nevertheless, it cannot be absolutely ruled out that some pupils were unable to participate in the programmes due to quarantine or the approach of half-class teaching during this time. From the academic year 2020/21 onwards, all the physical activities, as well as the open gym, were implemented regularly, although with precautions such as the wearing of a face mask and without activities that required physical contact.

## 5 Conclusions

In this study, the open gym proved to be a significant factor in promoting an active day for pupils. Theoretically, the open gym is an extended educational programme that is open to all pupils. For future studies, motor skills and sporting interests must be subject to more rigorous analysis. Do only pupils who are already interested in or competent at sports engage with the gym, or is the need to be active after a long period of being sedentary decisive for the choice, irrespective of motor skills? What does the introduction of an open gym imply for the future of all-day schools?

Important critical questions to ask in this context are; which additional activities should be offered in the interim, and if there is true equity in the decision-making process for pupils with regard to the use of the open gym. Additionally, during the open gym activity sessions, it is relevant to understand which pupils prevail in the negotiation processes and control the free play and whether all pupils have equal opportunities to participate. A key aspect of this programme is that it should inherently differ from other extended educational programmes and in particular from regular physical educational lessons, namely by offering space for extended learning opportunities centred on self-organization, initiative, participation, and autonomy (21). In order to investigate the long-term impact of the implementation of these programmes, further data collection should be carried out. This would elucidate whether the programs are attended on a sustained basis or whether the pupils' interest decreases over time.

Finally, this research indicates that the open gym demands of professionals an alternative style of management than that deployed within traditional classroom contexts. This challenges social pedagogues and educational trainers to work with an unstable and unpredictable number of pupils and to initiate and promote non-formal learning opportunities. Considering that both the ability and qualifications of staff can be understood as key factors in the effective delivery of all-day schooling (36), it will be necessary to develop and implement appropriate and effective training and further education that equips social pedagogues and trainers with the necessary competencies to implement the open gym effectively.

## Data Availability

The raw data supporting the conclusions of this article will be made available by the authors, without undue reservation.
